# Relationships between functional diversity and aboveground biomass production in the Northern Tibetan alpine grasslands

**DOI:** 10.1038/srep34105

**Published:** 2016-09-26

**Authors:** Juntao Zhu, Lin Jiang, Yangjian Zhang

**Affiliations:** 1 Lhasa Plateau Ecosystem Research Station, Key Laboratory of Ecosystem Network Observation and Modeling, Institute of Geographic Sciences and Natural Resources Research, Chinese Academy of Sciences, Beijing, 100101, China; 2 School of Biology, Georgia Institute of Technology, Atlanta, GA, 30332, USA; 3 CAS Center for Excellence in Tibetan Plateau Earth Sciences, Beijing 100101, China

## Abstract

Functional diversity, the extent of functional differences among species in a community, drives biodiversity–ecosystem function (BEF) relationships. Here, four species traits and aboveground biomass production (*ABP*) were considered. We used two community-wide measures of plant functional composition, (1) community weighted means of trait values (*CWM*) and (2) functional trait diversity based on Rao’s quadratic diversity (*FD*_*Q*_) to evaluate the effects of functional diversity on the *ABP* in the Northern Tibetan alpine grasslands. Both species and functional diversity were positively related to the *ABP*. Functional trait composition had a larger predictive power for the *ABP* than species diversity and *FD*_*Q*_, indicating a primary dependence of ecosystem property on the identity of dominant species in our study system. Multivariate functional diversity was ineffective in predicting ecosystem function due to the trade-offs among different traits or traits selection criterions. Our study contributes to a better understanding of the mechanisms driving the BEF relationships in stressed ecosystems, and especially emphasizes that abiotic and biotic factors affect the BEF relationships in alpine grasslands.

The rapid decline of global biodiversity has motivated considerable research directed towards understanding its potential consequences for ecosystem functioning^1^. While much work has focused on species diversity as an important dimension of biodiversity^2^, it has been increasingly recognized that functional diversity, the extent of functional differences among species in a community^3^, not the taxonomic richness, ultimately drives biodiversity–ecosystem functioning (BEF) relationships^4^,^5^,^6^. There is now extensive evidence that species traits are better at capturing the functional characteristics of a community and that the use of species traits in ecology significantly contributes to achieving a predictive framework for ecosystem functioning^7^,^8^,^9^,^10^.

Effects of functional traits on ecosystem properties have been quantified by two conceptually different approaches^11^. On the one hand, community-weighted means of trait values (*CWM*) are calculated as mean trait values weighted by species relative abundances in a community^12^, and are consequently closely related to the “mass ratio hypothesis”^13^, which proposes that ecosystem processes are strongly influenced by the functional traits of dominant species in a community. The *CWM* is therefore also linked to the sampling or selection effects associated with the greater chance of including highly productive species in more diverse communities^14^. On the other hand, a number of continuous measures have been developed which assess functional trait diversity of a community by quantifying the distribution of trait values among species^15^. Rao’s quadratic diversity *FD*_*Q*_ is the sum of pairwise functional distances between species weighted by their relative abundances. It reaches a maximum value when functionally different species, i.e. those with large trait differences, reach similarly high abundances^16^. Defined as such, *FD*_*Q*_ is related to facilitation and/or complementary resource use among species^17^.

Recent studies have shown that, *CWM*^18^,^19^, *FD*_*Q*_^20^,^21^, or a combination of *CWM* and *FD*_*Q*_^22^,^23^,^24^, can explain variation in ecosystem functioning. However, these results were mainly obtained by using experimentally created assemblages^25^,^26^, a method being criticized on the artificiality of the communities created^27^,^28^,^29^. In all, we still lack knowledge about relationships between functional diversity and ecosystem functioning for natural ecosystems, raising the question of whether the various species diversity-productivity relationships found in nature^30^ would apply to functional diversity.

Ecosystems are subject to natural environmental conditions with temporal and spatial variations, such as temperature, precipitation and nutrient availability^31^, as well as to influences from other species and human activities^25^,^32^. These effects vary in their frequencies and intensities, including regularly recurring variations to which organisms living in a given environment are more or less adapted, and episodic, catastrophic disturbances that lead to extensive mortality and local species extinction^33^. Currently, a few studies^34^,^35^,^36^ have been done in BEF relationships under these stressful conditions. The small number of studies on this topic have reported contrasting results, ranging from clearly positive to no or in some circumstances even negative effects of diversity on ecosystem functioning under environmental stress^37^.

In the Northern Tibetan plateau, there are three main natural vegetation types, alpine meadow, alpine steppe and alpine desert steppe^38^, and most of plants are perennial herbs. The vegetation in this area is exposed to extreme environmental conditions, including intense radiation, strong winds, low temperatures, low soil nutrients and drought stress^38^,^39^. The northern Tibetan ecosystem is an ideal site to evaluate relationships between functional diversity and ecosystem functioning for natural ecosystems under stressful conditions. In this paper, we selected four species traits, plant height (*H*), plant coverage (*C*), leaf mass per area (*LMA*), leaf dry matter content (*LDMC*), and aboveground biomass production (*ABP*). Firstly, we tested the hypotheses that functional diversity could explain more variation in ecosystem functioning than species diversity. Secondly, we related aboveground biomass production and trait-based indices to assess whether *CWM*, i.e. functional identity of dominant species, or *FD*_*Q*_, i.e. functional dissimilarity among species, are better predictors for ecosystem functioning.

## Materials and Methods

### Study sites

**Figure 1:**
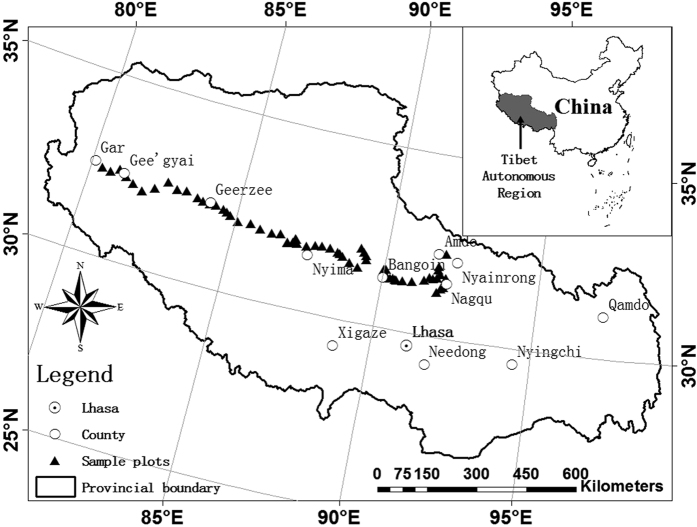
Distribution of the 63 study sites along the Northern Tibetan Plateau Alpine Grassland Transect. The national and county boundary datasets in shapfile format were downloaded from the National Geomatics Center of China (http://www.ngcc.cn/) freely. This figure was exported in a JPEG format from ArcGIS 9.3 software (http://www.esri.com/software/arcgis/index.html).

The Northern Tibetan Plateau (locally named Changtang) is located in the hinterland of the Qinghai–Tibet Plateau (29°53′–36°32′ N; 78°41′–92°16′E), covering an area of 597,000 km^2^ (Fig. 1). We set up a west–east alpine grassland transect (the Northern Tibetan Plateau Alpine Grassland Transect, NTPAGT) in May 2009^40^. The NTPAGT covers longitudes from 79.71 to 92.03° E and latitudes from 30.50 to 33.45° N, and was approximately 1, 200 km long and 400 km wide^38^. The mean annual precipitation (MAP) decreases from east of 550 mm to west of 60 mm and mean annual temperature (MAT) ranges from −2.3 °C to 1.2 °C (a more sophisticated description of the environmental settings see the climate diagrams in Supplement, Figs S1 and S2). The elevation of the sample sites ranges from 4374 to 4953 m. The growing season in this region usually begins in May and ends in September, with 65 to 85% of precipitation occurring during this period. The zonal alpine grassland types and their aboveground biomass production follow the general climate pattern^41^.

The NTPAGT traverses three main natural vegetation types: alpine meadow, alpine steppe and alpine desert steppe^38^. Vegetation in alpine meadow is dominated by *Kobresia pygmaea*, associated with *Potentilla saundersiana, Potentilla cuneata, Stipa purpurea* and *Festuca coelestis*. The alpine meadow is located in the eastern Changtang, where an alpine semi-humid climate dominates. Alpine steppe, dominated by *S. purpurea, Stipa capillacea* and *Stipa subsessiliflora* var. *basiplumosa*, associated with *Kobresia humilis, Carex moorcroftii, Leontopodium nanum, Oxytropis microphylla*. The alpine steppe is widely distributed in the middle Changtang, where an alpine semi-arid climate dominates. Alpine desert steppe (dominated by *S. purpurea, Ceratoides lateens* and *Stipa glareosa*) is scattered across the western and northwestern Changtang, where the climate is an alpine arid type^40^.

### Species abundance, aboveground biomass production and leaf traits

Field surveys were conducted during late July to early August in 2011 and 2012. Sixty-two fenced sites (310 plant quadrats) were surveyed one time across the growing season along the transect (Fig. 1). There were 18, 28 and 16 sites in the alpine meadow, alpine steppe and alpine desert steppe system, respectively. We recorded geographical coordinates, elevation, and vegetation type for each site. Five 1 m × 1 m quadrats were laid out randomly within each 100 m × 100 m site and all vascular plant species were recorded. The number of species and individual plants (genets and ramets), individual plant height, coverage and weight, and canopy coverage were measured in each quadrat. We harvested the aboveground biomass down to the soil surface and sorted the individuals by species. The major sampled species reached peak coverage usually during the field period (late July to early August). Therefore, the aboveground biomass in this region can serve as a surrogate for ANPP^42^. Aboveground biomass was weighed after removing dead parts and being oven–dried at 65 °C for 72 hours to a constant weight.

According to the standard measurement methods of plant traits^43^, we selected four plant traits associated with productivity: plant height (*H*), plant coverage (*C*), leaf mass per area (*LMA*) and leaf dry matter content (*LDMC*). Five dominant species in each quadrat were measured, with the biomass of these species accounting for more than 80% of the total biomass. Twenty intact leaves were randomly selected for each species. We scanned the leaf area, and oven–dried leaves at 65 °C for 72 hours to a constant weight. Leaf area was calculated using Sigmascan 4.1.

### Functional diversity and Shannon-Weaver index

The community-weighted mean trait values (*CWM*) for each trait were calculated for each plant quadrat (n = 310), following Garnier *et al*.^18^:


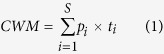


where *S* is the number of species in a community, *p*_*i*_ is the relative abundance of species *i* and the biomass for each species as the relative abundance, *t*_*i*_ is the species-specific trait value.

The Rao’s functional diversity index (*FD*_*Q*_) was calculated using the Excel-macro developed by Lepš *et al*.^44^ according to the equation:


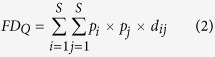


where *S* is the number of species in a community, *p*_*i*_ and *p*_*j*_ are the relative abundances of species *i* and *j*, and *d*_*ij*_ is the trait distance between species *i* and *j* in a community.

The Shannon-Weaver index (*H*) was calculated for each community using the equation (Shannon & Weaver 1949)^45^:


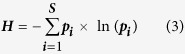


where *S* is the number of species in a community, *p*_*i*_ is the relative abundances of species.

Single- and multi-trait indices contained unique information about functional composition of the communities, and both are likely to have a place in predicting variations in ecosystem functions under different scenarios^46^. So we also calculated multi-trait functional diversity as functional dispersion (*FD*is). In multidimensional trait space, *FD*is is the mean distance of each species, weighted by its relative abundances, to the centroid of all species in a community^47^. *CWM, FD*_*Q*_ for each trait individually and *FD*is for all traits in combination were calculated using the FD package^48^ in R version 3.1.1 (R Development Core Team 2014).

### Statistical analysis

Data on species traits, species diversity and aboveground biomass production were log transformed prior to analysis to meet the assumptions of normality. A general linear regression analyses was used to examine relationships between diversity and aboveground biomass production. According to the major plant composition, we divided our 310 sample plots into five plant functional groups, including (group I, 57 plots) *Stipa purpurea* - *Stipa subsessiflora - Carex moorcroftii*; (group II, 63 plots) *Stipa purpurea - Artemisia duthreuil-de-rhinsi*; (group III, 70 plots) *Stipa purpurea - Carex moorcroftii*; (group IV, 60 plots) *Kobresia pygmaea* - *Stipa purpurea* - *Leontopodium ochroleucum*; (group V, 60 plots) *Kobresia pygmaea* - *Potentilla saundersiana*, respectively (Table 1). The mean annual precipitation (MAP) of five groups is 60–150, 150–260, 280–360, 360–440, and 440–550 mm, respectively. We assess the BEF relationships for each plant functional group. Multiple stepwise regression method was used to determine the major traits affecting ecosystem functions. All statistical analyses were performed using the R statistical package version 3.1.1 (R Core Team 2014).

## Results

**Figure 2:**
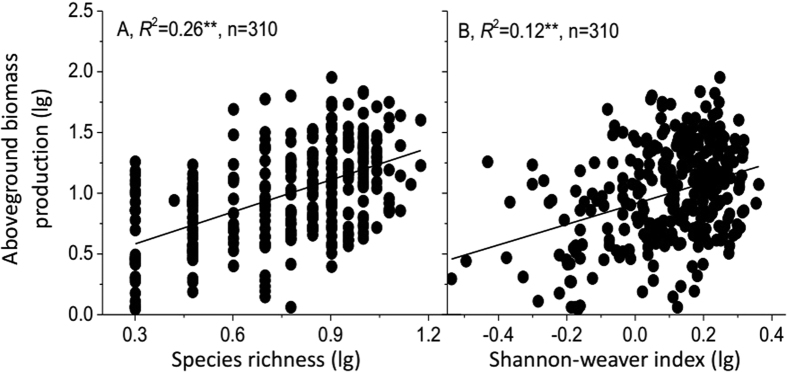
Relationships between species richness (**A**), Shannon-Weaver index (**B**) and aboveground biomass production. Lines show the fitted lg–lg relationships. n is the sample size (number of plots). Asterisks indicate significance: ^**^P < 0.001; ^*^P < 0.01; NS = not significant.

**Figure 3:**
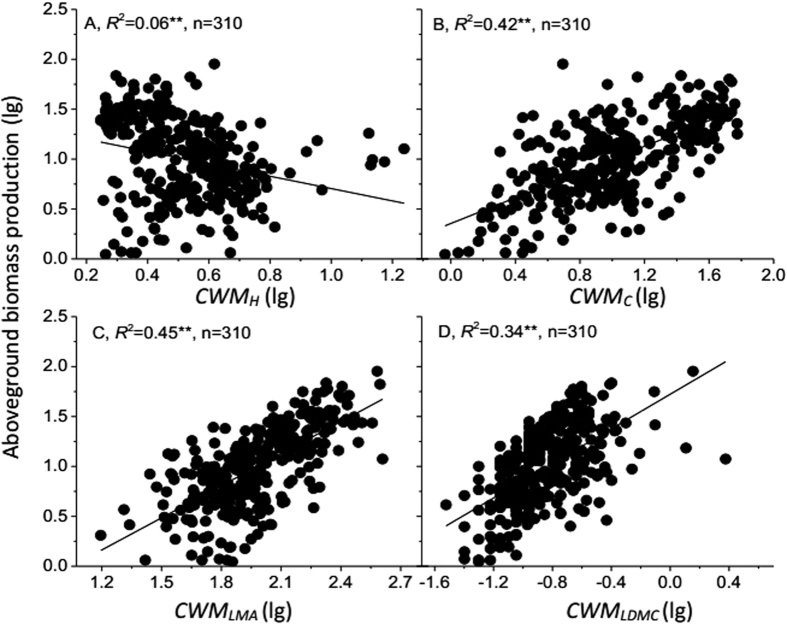
Relationships between community-weighted means of four traits (*CWM*_*H*_, *CWM*_*C*_, *CWM*_*LMA*_ and *CWM*_*LDMC*_; (**A–D**) and aboveground biomass production. Lines show the fitted lg–lg relationships. *CWM*_*H*_, *CWM*_*C*_, *CWM*_*LMA*_ and *CWM*_*LDMC*_ indicate community-weighted means of plant height, plant coverage, leaf mass per area and leaf dry matter content, respectively. n is the sample size (number of plots). Asterisks indicate significance levels: ^**^P < 0.001; ^*^P < 0.01; NS = not significant.

For the Northern Tibetan alpine grasslands, aboveground biomass production (*ABP*) significantly increased with species richness (*SR*) and Shannon-Weaver index (*H*) (*P* < 0.001, Fig. 2). *The ABP* significantly increased with community**-**weighted means of plant coverage (*CWM*_*C*_), leaf mass per area (*CWM*_*LMA*_) and leaf dry matter content (*CWM*_*LDMC*_) (*P* < 0.001), but significantly decreased with community-weighted means of plant height (*CWM*_*H*_) (*P* < 0.001, Fig. 3). *CWM*_*LMA*_, *CWM*_*C*_and *CWM*_*LDMC*_ had a higher explanatory power on the *ABP* than *CWM*_*H*_ (Fig. 3). Multiple regression analyses showed that the ABP mainly depended on *CWM*_*LMA*_, *CWM*_*C*_and *CWM*_*LDMC*_ (*P* < 0.001, *R*^2^ = 0.82, Table 2). *CWM* explained a larger proportion of variation in the *ABP* than did *SR* and *H*, except for *CWM*_*H*_(Figs 2 and 3).

**Figure 4:**
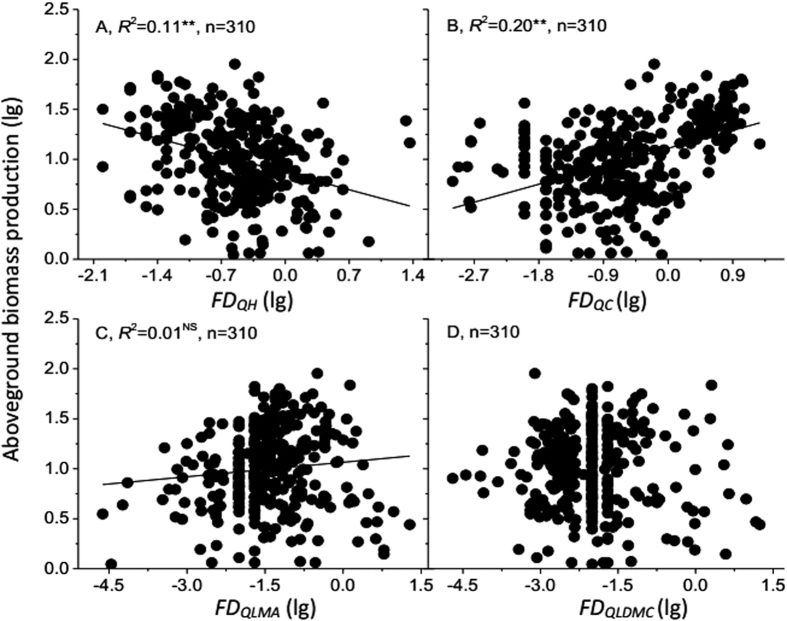
Relationships between Rao’s functional diversity index of four traits (*FD*_*QH*_, *FD*_*QC*_, *FD*_*QLMA*_ and *FD*_*QLDMC*_; (**A–D**) and aboveground biomass production. Lines show the fitted lg–lg relationships. *FD*_*QH*_, *FD*_*QC*_, *FD*_*QLMA*_ and *FD*_*QLDMC*_ indicate Rao’s functional diversity index of plant height, plant coverage, leaf mass per area and leaf dry matter content, respectively. n is the sample size (number of plots). Asterisks indicate significance level: ^**^P < 0.001; ^*^P < 0.01; NS = not significant.

**Figure 5:**
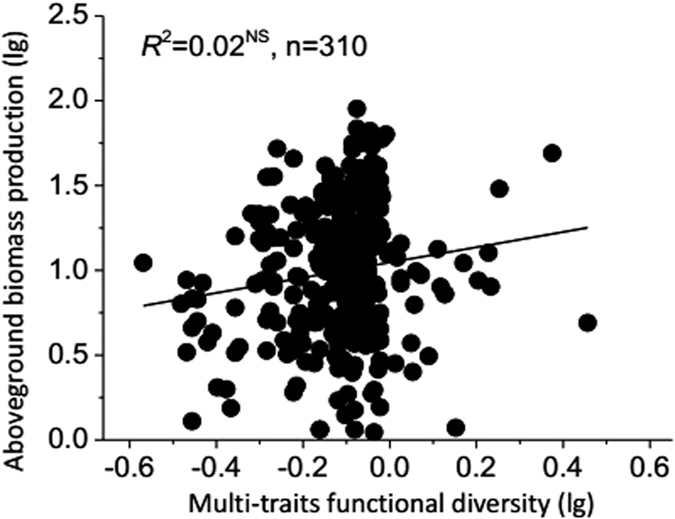
Relationships between multivariate functional diversity of plant height, plant coverage, leaf mass per area, leaf dry matter content and aboveground biomass production. n is the sample size (number of plots). Asterisks indicate significance level: ^**^P < 0.001; ^*^P < 0.01; NS = not significant.

*The ABP* significantly decreased with Rao’s functional diversity index of plant height (*FD*_*QH*_) (*P* < 0.001, Fig. 4A), but significantly increased with Rao’s index of plant coverage (*FD*_*QC*_) (*P* < 0.001, Fig. 4B). However, *FD*_*Q*_ did not explain a larger amount of variation in the *ABP* (20% and 11%) than *SR* and *H* (26% and 12% of explained variation respectively). No significant relationships were found between Rao’s index of leaf mass per area (*FD*_*QLMA*_), leaf dry matter content (*FD*_*QLDMC*_) and the *ABP* (Fig. 4C,D). When all traits were considered together, no significant relationships between functional diversity and ecosystem functions were identified (Fig. 5).

For different plant functional groups, the *ABP* significantly increased with *SR* in group I, II and IV (*P* < 0.001), but no significant relationships were found in group III and V (Fig. S3). *The ABP* in group II significantly increased with *H (P* < 0.001), but no significant relationships were found in other groups (Fig. S4). *The ABP* significantly increased with *CWM*_*H*_ in group I (*P* < 0.001), but significantly decreased in group III and IV (*P* < 0.001) and no significant relationships in group II and IV (Fig. S5). *The ABP* significantly increased with *CWM*_*C*_ in group I, III and IV (*P* < 0.001), but no significant relationships in group II and V (Fig. S6). *The ABP* significantly increased with *CWM*_*LMA*_ in group II, III, IV and V (*P* < 0.001), but no significant relationships in group I (Fig. S7). *The ABP* significantly increased with *CWM*_*LDMC*_ in all five groups (*P* < 0.001, Fig. S8). No significant relationships between *FD*_*Q*_ and the *ABP* were identified for most of the groups (Figs S9–12). For group I, II and V, *CWM*_*LDMC*_ had a higher explanatory power on the *ABP* than other three traits (Fig. S8A,B and E). For group III and IV, *CWM*_*LMA*_ explained a larger proportion of variation in the *ABP* than other three traits (Fig. S7C,D). Multiple regression analyses showed that the *ABP* in group I, II and V mainly depended on *CWM*_*LDMC*_and *CWM*_*C*_, and the *ABP* in group III and IV mainly depended on *CWM*_*LMA*_ and *CWM*_*C*_(Table 2).

## Discussion

It is widely reported that species diversity affects ecosystem functioning^2^. Consistent with this general pattern, we found a positive relationship between species richness (*SR*), Shannon-Weaver index (*H*) and aboveground biomass production (*ABP*). Our study also demonstrated that community-weighted mean values (*CWM*) of species traits explain a larger proportion of variation in the *ABP* than *SR* and *H*, thereby extending the results of previous related studies^4^,^5^,^6^ to the alpine grassland ecosystem. Collectively, we further evidenced that functional diversity, not the taxonomic richness, ultimately drives BEF relationships^3^. Alpine habitats are well known for their severe physical living conditions^49^, where plants are confronted by low temperatures, excessive radiation, strong winds, low soil nutrients, unstable substrates and short growing seasons^38^,^39^. In accord with previous findings^34^,^35^, our study indicated that both species diversity and functional diversity were positively related to ecosystem property, and evidenced positive effects of biodiversity on ecosystem functioning under stressful conditions.

We examined relationships between functional diversity based on *CWM, FD*_*Q*_ and ecosystem properties in natural alpine grasslands. Our results showed that the variation of *ABP* is better explained by *CWM*, indicating a primary dependence of ecosystem properties on the identity of dominant species and their functional traits following the ‘mass ratio hypothesis’^13^,^19^ in alpine grasslands. Indeed, functional identity attributed to the selection effect^14^, usually expressed as *CWM* for species traits, have been demonstrated to be a key predictor of ecosystem functioning at different scales^18^,^22^. Regardless of the varying dominant species in each grassland type and functional group, coverage and biomass of the dominant species account for 40–80% of the total in each community (sample plots data), which lends further support on the above conclusion.

Beyond functional identity, functional diversity (*FD*_*Q*_) has been shown to be associated with ecosystem functioning^25^,^50^, indicating the importance of niche complementarity in facilitating ecosystem processes^51^. However, our analyses did not find that *FD*_*Q*_ explain a larger amount of variation in the *ABP* than *SR* and *H*, suggesting a lack of the operation of the complementarity effects in our study system. In our study area, soil moisture and nutrients are in shortage for belowground plant parts^52^, which may have led to strong competitive interactions for below-ground resources (nitrogen and water), resulting in weakened complementary resource use among plant species.

For the alpine grasslands, species traits, e.g., leaf mass per area (*LMA*), plant coverage (*C*) and leaf dry matter content (*LDMC*) significantly contribute to achieving a predictive framework for ecosystem functioning^7^,^8^,^9^,^10^. Specifically, our analyses identified *LMA*, which is related to resource acquisition and plant growth strategy^53^,^54^, as a key functional predictor of ecosystem functioning. Our results were in accordance with previous studies suggesting that *LMA* can be used to predict productivity and carbon storage^11^,^55^. The leaf economic spectrum framework predicts that low *LMA* should promote productivity^7^. However, alpine grasslands had their maximum *ABP* at high *LMA*. Plants with high *LMA* could be adapted to low temperatures and high irradiance, and tend to have thick, leathery leaves^56^,^57^. Although plant height has been used to predict root/shoot ratio in alpine grasslands^58^, our results found that there was no significant relationship between plant height and ecosystem functioning.

For different plant functional groups, such as group II and IV, community-weighted means of leaf dry matter content (*CWM*_*LDMC*_) and leaf mass per area (*CWM*_*LMA*_), plant coverage (*CWM*_*C*_) significantly contribute to achieving a predictive framework for the *ABP* in group II (degraded alpine steppe) and group IV (degraded alpine meadow), respectively. Hence, we should selected the dominant species with higher leaf dry matter content (*LDMC*) to maintain high productivity for restoring degenerative alpine steppe. For the degraded alpine meadow, the dominant species with higher leaf mass per area (*LMA*) and plant coverage (*C*) should be selected to maintain high productivity.

When all traits were considered together (multivariate functional diversity), no significant relationships between functional diversity and ecosystem functions were identified in this study. This result supports that in a complex landscape with multiple environmental gradients, for example our alpine grassland transect, including three vegetation types and multiple gradients (precipitation, elevation, and soil nutrients, etc.), variation in a single trait can explain more variation in ecosystem functioning than functional diversity calculated based on multiple traits^46^. In addition, plant traits are positively (e.g. leaf mass per area) or negatively (e.g. plant height) related to the *ABP*, that is trade-offs among different traits could render diverse communities less capable of providing multiple functional diversity in our study. Therefore, the trade-offs among different traits or traits selection criterions must be considered when multivariate functional diversity is used to predict ecosystem function.

## Additional Information

**How to cite this article**: Zhu, J. *et al*. Relationships between functional diversity and aboveground biomass production in the Northern Tibetan alpine grasslands. *Sci. Rep.* **6**, 34105; doi: 10.1038/srep34105 (2016).

## Supplementary Material

Supplementary Information

## Figures and Tables

**Table 1:** According to the major plant composition, we divided 310 sample plots into five plant functional groups.

Groups	Plant composition	Grassland types	Altitude (m)	Sample number	Longitude (°)	Latitude (°)
Group I	*Stipa purpurea* - *Stipa subsessiflora - Carex moorcroftii*	Alpine desert steppe	4374–4814	57	80.38–84.05	32.08–32.51
Group II	*Stipa purpurea - Artemisia duthreuil-de-rhinsi*	Degraded alpine steppe	4435–4953	63	84.26–87.09	31.75–32.26
Group III	*Stipa purpurea - Carex moorcroftii*	Alpine steppe	4533–4803	70	86.80–90.08	31.50–32.18
Group IV	*Kobresia pygmaea* - *Stipa purpurea* - *Leontopodium ochroleucum*	Degraded alpine meadow	4548–4788	60	89.35–91.72	31.36–31.86
Group V	*Kobresia pygmaea* - *Potentilla saundersiana*	Alpine meadow	4537–4788	60	91.72–92.01	31.22–31.94

**Table 2:** Stepwise regression equations of community aboveground biomass production (*ABP*) and species diversity, functional diversity.

Groups	Regression equations	*R*^2^	*P*
Total	*ABP* = −0.346 + 0.609*CWM*_*LMA*_ + 0.565*CWM*_*C*_ + 0.503*CWM*_*LDMC*_	0.82	*P* < 0.001
Group I	*ABP* = 0.887 + 0.615*CWM*_*LDMC*_ + 0.459*CWM*_*C*_	0.73	*P* < 0.001
Group II	*ABP* = 1.715 + 1.084*CWM*_*LDMC*_	0.79	*P* < 0.001
Group III	*ABP* = −1.651 + 1.077*CWM*_*LMA*_ + 0.506*CWM*_*C*_	0.76	*P* < 0.001
Group IV	*ABP* = −1.557 + 1.013*CWM*_*LMA*_ + 0.534*CWM*_*C*_	0.75	*P* < 0.001
Group V	*ABP* = 1.722 + 0.903*CWM*_*LDMC*_ + 0.274*CWM*_*C*_	0.76	*P* < 0.001

*CWM*_*C*_, *CWM*_*LMA*_ and *CWM*_*LDMC*_, *indicated* community weighted means of plant coverage, leaf mass per area, leaf dry matter content, respectively. Group I, II, III, IV, V, indicate five plant functional groups, respectively.
