# Supplementary material for: Relationships between functional diversity and aboveground biomass production in the Northern Tibetan alpine grasslands
Source: Sci Rep. 2016 Sep 26;6:34105. doi: 10.1038/srep34105 (PMC5036173; doi:10.1038/srep34105)
Supplement: Supplementary Information [file srep34105-s1.doc]

**Relationships between functional diversity and aboveground biomass production** **in the Northern Tibetan alpine grasslands**

**Juntao Zhu^1*^, Lin Jiang^2^, Yangjian Zhang^1, 3^**

^1^*Lhasa Plateau Ecosystem Research Station, Key Laboratory of Ecosystem Network Observation and Modeling,* *Institute of Geographic Sciences and Natural Resources Research, Chinese Academy of Sciences, Beijing, 100101, China*

^2^*School of Biology, Georgia Institute of Technology,* *Atlanta, GA,* *30332, USA*

^3^*CAS Center for Excellence in Tibetan Plateau Earth Sciences, Beijing 100101, China*

Juntao Zhu, *Correspondence to E-mail: [zhujt@igsnrr.ac.cn](mailto:zhujt@igsnrr.ac.cn)

Lin Jiang, E-mail: [lin.jiang@biology.gatech.edu](mailto:lin.jiang@biology.gatech.edu)

Yangjian Zhang, E-mail: [zhangyj@igsnrr.ac.cn](mailto:zhangyj@igsnrr.ac.cn)


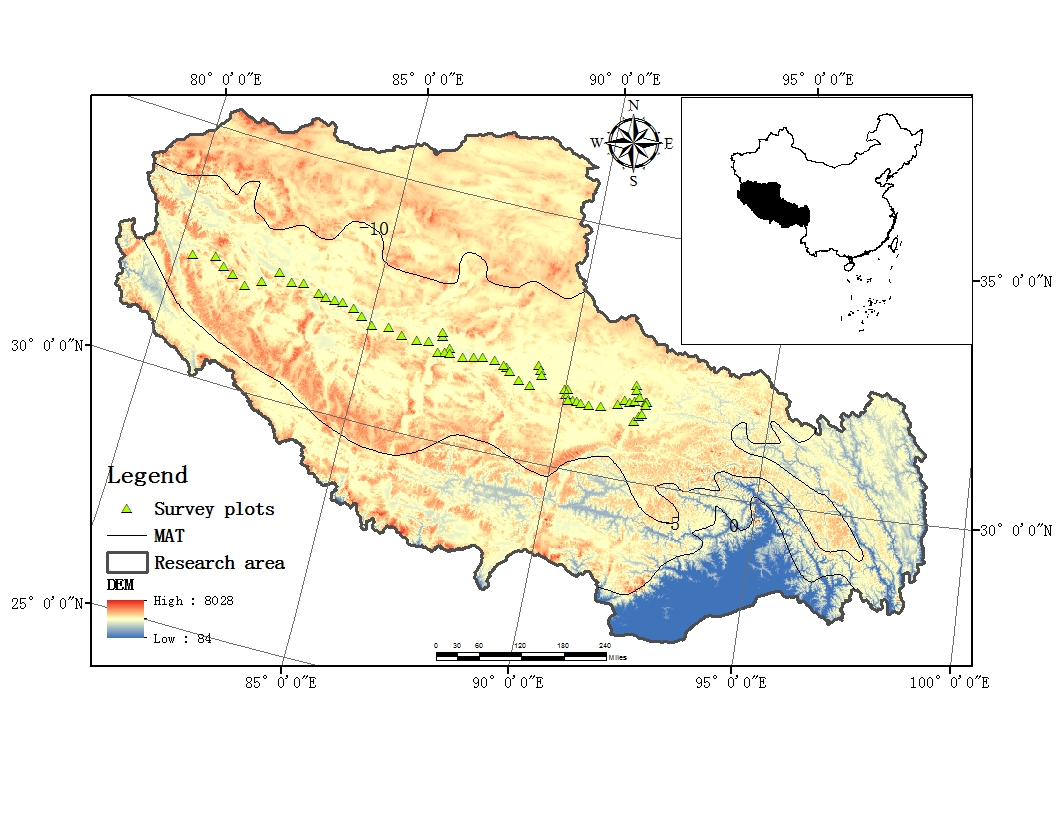


**Figure. S1** Spatial pattern of mean annual temperature from 1982 to 2013 on the Northern Tibetan Plateau Alpine Grassland Transect. Monthly temperature data obtained from the National Meteorological Information Centre of China Meteorological Administration were interpolated by Anusplin software (AUSTRALIAN NATIONAL UNIVERSITY, 4.2). The national and county boundary datasets in shapfile format were downloaded from the National Geomatics Center of China (<http://www.ngcc.cn/>) freely. This figure was  exported in a JPEG format from ArcGIS 9.3 software (<http://www.esri.com/software/arcgis/index.html>).


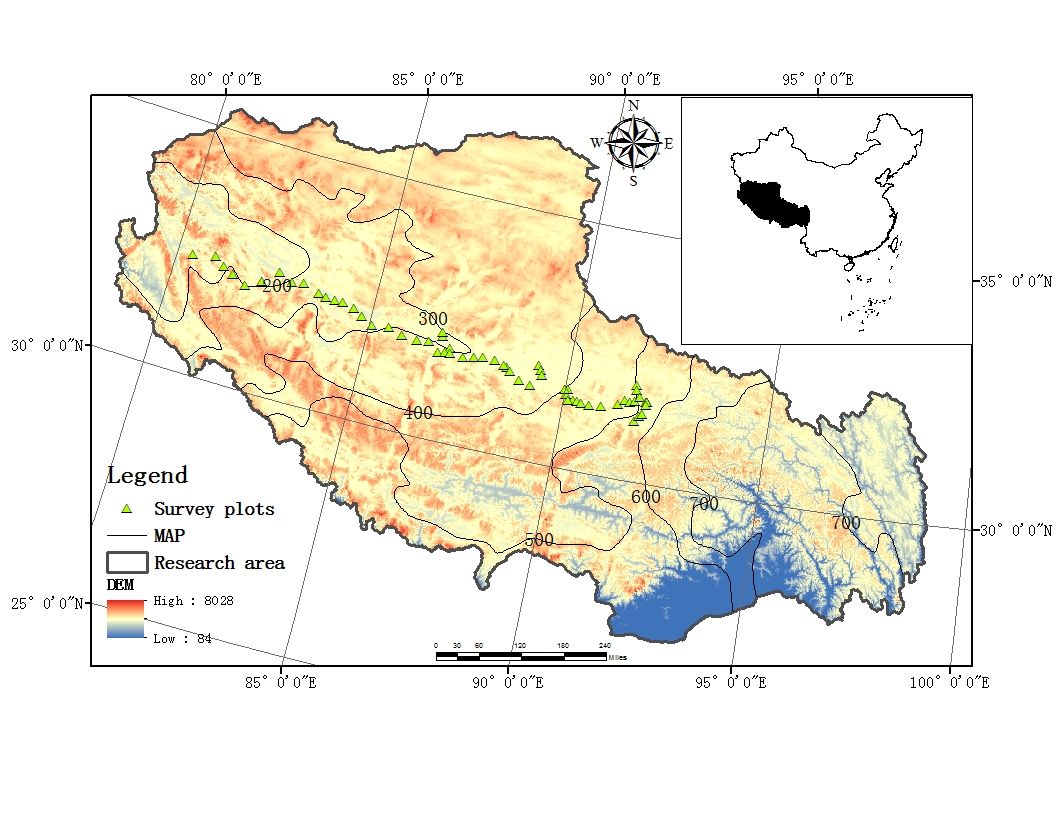


**Figure. S2** Spatial pattern of mean annual precipitation from 1982 to 2013 on the Northern Tibetan Plateau Alpine Grassland Transect. Monthly precipitation data obtained from the National Meteorological Information Centre of China Meteorological Administration were interpolated by Anusplin software (AUSTRALIAN NATIONAL UNIVERSITY, 4.2). The national and county boundary datasets in shapfile format were downloaded from the National Geomatics Center of China (<http://www.ngcc.cn/>) freely. This figure was  exported in a JPEG format from ArcGIS 9.3 software (http://www.esri.com/software/arcgis/index.html).

Aboveground biomass production (lg)

Species richness (lg)

**Figure S3.** Relationships between species richness and aboveground biomass production among different plant functional groups. (A) *Stipa purpurea* - *Stipa subsessiliflom - Carex moorcroftii*; (B) *Stipa purpurea - Artemisia duthreuil-de-rhinsi*; (C) *Stipa purpurea - Carex moorcroftii*; (D) *Kobresia pygmaea* - *Stipa purpurea* - *Leontopodium ochroleucum*; (E) *Kobresia pygmaea* - *Potentilla saundersiana*. Lines show the fitted lg–lg relationships. n is the sample size (number of plots). Asterisks indicate significance: ^**^ = *P* < 0.001; ^*^ = *P* < 0.01; ^NS^ = not significant.

Aboveground biomass production (lg)

Shannon-Weaver index (lg)

**Figure S4.** Relationships between Shannon-Weaver index and aboveground biomass production among different plant functional groups. (A) *Stipa purpurea* - *Stipa subsessiliflom - Carex moorcroftii*; (B) *Stipa purpurea - Artemisia duthreuil-de-rhinsi*; (C) *Stipa purpurea - Carex moorcroftii*; (D) *Kobresia pygmaea* - *Stipa purpurea* - *Leontopodium ochroleucum*; (E) *Kobresia pygmaea* - *Potentilla saundersiana*. Lines show the fitted lg–lg relationships. n is the sample size (number of plots). Asterisks indicate significance: ^**^ = *P* < 0.001; ^*^ = *P* < 0.01; ^NS^ = not significant.

Aboveground biomass production (lg)

*CWM_H_* (lg)

**Figure S5.** Relationships between community-weighted means of plant height (*CWM_H_*) and aboveground biomass production among different plant functional groups. (A) *Stipa purpurea* - *Stipa subsessiliflom - Carex moorcroftii*; (B) *Stipa purpurea - Artemisia duthreuil-de-rhinsi*; (C) *Stipa purpurea - Carex moorcroftii*; (D) *Kobresia pygmaea* - *Stipa purpurea* - *Leontopodium ochroleucum*; (E) *Kobresia pygmaea* - *Potentilla saundersiana*. Lines show the fitted lg–lg relationships. n is the sample size (number of plots). Asterisks indicate significance: ^**^ = *P* < 0.001; ^*^ = *P* < 0.01; ^NS^ = not significant.

Aboveground biomass production (lg)

*CWM_C_* (lg)

**Figure S6.** Relationships between community-weighted means of plant coverage (*CWM_C_*) and aboveground biomass production among different plant functional groups. (A) *Stipa purpurea* - *Stipa subsessiliflom - Carex moorcroftii*; (B) *Stipa purpurea - Artemisia duthreuil-de-rhinsi*; (C) *Stipa purpurea - Carex moorcroftii*; (D) *Kobresia pygmaea* - *Stipa purpurea* - *Leontopodium ochroleucum*; (E) *Kobresia pygmaea* - *Potentilla saundersiana*. Lines show the fitted lg–lg relationships. n is the sample size (number of plots). Asterisks indicate significance: ^**^ = *P* < 0.001; ^*^ = *P* < 0.01; ^NS^ = not significant.

Aboveground biomass production (lg)

*CWM_LMA_* (lg)

**Figure S7.** Relationships between community-weighted means of leaf mass per area (*CWM_LMA_*) and aboveground biomass production among different plant functional groups. (A) *Stipa purpurea* - *Stipa subsessiliflom - Carex moorcroftii*; (B) *Stipa purpurea - Artemisia duthreuil-de-rhinsi*; (C) *Stipa purpurea - Carex moorcroftii*; (D) *Kobresia pygmaea* - *Stipa purpurea* - *Leontopodium ochroleucum*; (E) *Kobresia pygmaea* - *Potentilla saundersiana*. Lines show the fitted lg–lg relationships. n is the sample size (number of plots). Asterisks indicate significance: ^**^ = *P* < 0.001; ^*^ = *P* < 0.01; ^NS^ = not significant.

Aboveground biomass production (lg)

*CWM_LDMC_* (lg)

**Figure S8.** Relationships between community-weighted means of leaf dry matter content (*CWM_LDMC_*) and aboveground biomass production among different plant functional groups. (A) *Stipa purpurea* - *Stipa subsessiliflom - Carex moorcroftii*; (B) *Stipa purpurea - Artemisia duthreuil-de-rhinsi*; (C) *Stipa purpurea - Carex moorcroftii*; (D) *Kobresia pygmaea* - *Stipa purpurea* - *Leontopodium ochroleucum*; (E) *Kobresia pygmaea* - *Potentilla saundersiana*. Lines show the fitted lg–lg relationships. n is the sample size (number of plots). Asterisks indicate significance: ^**^ = *P* < 0.001; ^*^ = *P* < 0.01; ^NS^ = not significant.

Aboveground biomass production (lg)

*FD_QH_* (lg)

**Figure S9.** Relationships between Rao´s functional diversity index of plant height (*FD_QH_*) and aboveground biomass production among different plant functional groups. (A) *Stipa purpurea* - *Stipa subsessiliflom - Carex moorcroftii*; (B) *Stipa purpurea - Artemisia duthreuil-de-rhinsi*; (C) *Stipa purpurea - Carex moorcroftii*; (D) *Kobresia pygmaea* - *Stipa purpurea* - *Leontopodium ochroleucum*; (E) *Kobresia pygmaea* - *Potentilla saundersiana*. Lines show the fitted lg–lg relationships. n is the sample size (number of plots). Asterisks indicate significance: ^**^ = *P* < 0.001; ^*^ = *P* < 0.01; ^NS^ = not significant.

Aboveground biomass production (lg)

*FD_QC_* (lg)

**Figure S10.** Relationships between Rao´s functional diversity index of plant coverage (*FD_QC_*) and aboveground biomass production among different plant functional groups. (A) *Stipa purpurea* - *Stipa subsessiliflom - Carex moorcroftii*; (B) *Stipa purpurea - Artemisia duthreuil-de-rhinsi*; (C) *Stipa purpurea - Carex moorcroftii*; (D) *Kobresia pygmaea* - *Stipa purpurea* - *Leontopodium ochroleucum*; (E) *Kobresia pygmaea* - *Potentilla saundersiana*. Lines show the fitted lg–lg relationships. n is the sample size (number of plots). Asterisks indicate significance: ^**^ = *P* < 0.001; ^*^ = *P* < 0.01; ^NS^ = not significant.

Aboveground biomass production (lg)

*FD_QLMA_* (lg)

**Figure S11.** Relationships between Rao´s functional diversity index of leaf mass per area (*FD_QLMA_*) and aboveground biomass production among different plant functional groups. (A) *Stipa purpurea* - *Stipa subsessiliflom - Carex moorcroftii*; (B) *Stipa purpurea - Artemisia duthreuil-de-rhinsi*; (C) *Stipa purpurea - Carex moorcroftii*; (D) *Kobresia pygmaea* - *Stipa purpurea* - *Leontopodium ochroleucum*; (E) *Kobresia pygmaea* - *Potentilla saundersiana*. Lines show the fitted lg–lg relationships. n is the sample size (number of plots). Asterisks indicate significance: ^**^ = *P* < 0.001; ^*^ = *P* < 0.01; ^NS^ = not significant.

Aboveground biomass production (lg)

*FD_QLDMC_* (lg)

**Figure S12.** Relationships between Rao´s functional diversity index of leaf dry matter content (*FD_QLDMC_*) and aboveground biomass production among different plant functional groups. (A) *Stipa purpurea* - *Stipa subsessiliflom - Carex moorcroftii*; (B) *Stipa purpurea - Artemisia duthreuil-de-rhinsi*; (C) *Stipa purpurea - Carex moorcroftii*; (D) *Kobresia pygmaea* - *Stipa purpurea* - *Leontopodium ochroleucum*; (E) *Kobresia pygmaea* - *Potentilla saundersiana*. Lines show the fitted lg–lg relationships. n is the sample size (number of plots). Asterisks indicate significance: ^**^ = *P* < 0.001; ^*^ = *P* < 0.01; ^NS^ = not significant.
